# Supplementary material for: Direct Mercury Detection in Landfill Leachate Using a Novel AuNP-Biopolymer Carbon Screen-Printed Electrode Sensor
Source: Micromachines (Basel). 2021 Jun 1;12(6):649. doi: 10.3390/mi12060649 (PMC8229311; doi:10.3390/mi12060649)
Supplement: Supplementary file 1 [file micromachines-12-00649-s001.zip › micromachines-1230272-supplementary.pdf]

**Supporting Information**

# Direct mercury detection in landfill leachate using a novel AuNP-biopolymer carbon screen-printed electrode sensor

Jae-Hoon Hwang [1,*], David Fox [2], Jordan Stanberry [3], Vasileios Anagnostopoulos [3], Lei Zhai [2] and Woo Hyoung Lee [1,*]

[1] Department of Civil, Environmental, and Construction Engineering, University of Central Florida, Orlando, FL, 32816, USA
[2] NanoScience Technology Center and Department of Chemistry, University of Central Florida, Orlando, FL, 32816, USA
[3] Department of Chemistry, University of Central Florida, Orlando, FL, 32816, USA

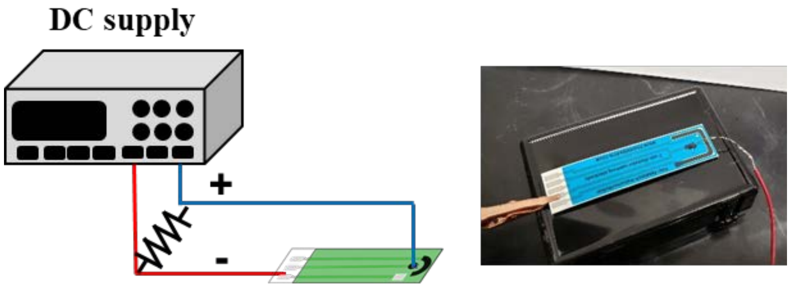


**Figure S1.** A schematic of the AuNP-biopolymer coated carbon SPE sensor fabrication process (electrodeposition).

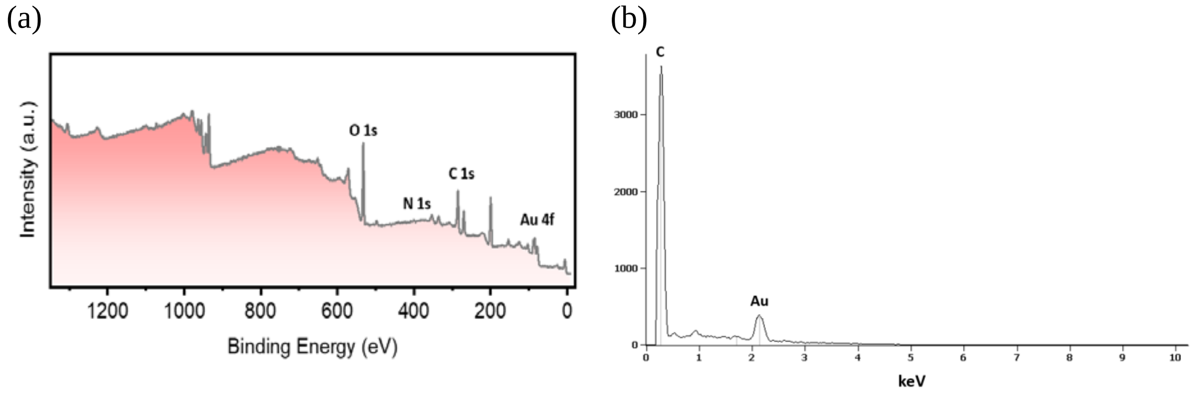


**Figure S2.** Characterization of an AuNP-biopolymer coated carbon SPE sensor. **(a)** Survey peak of XPS and **(b)** EDS spectrum of SEM

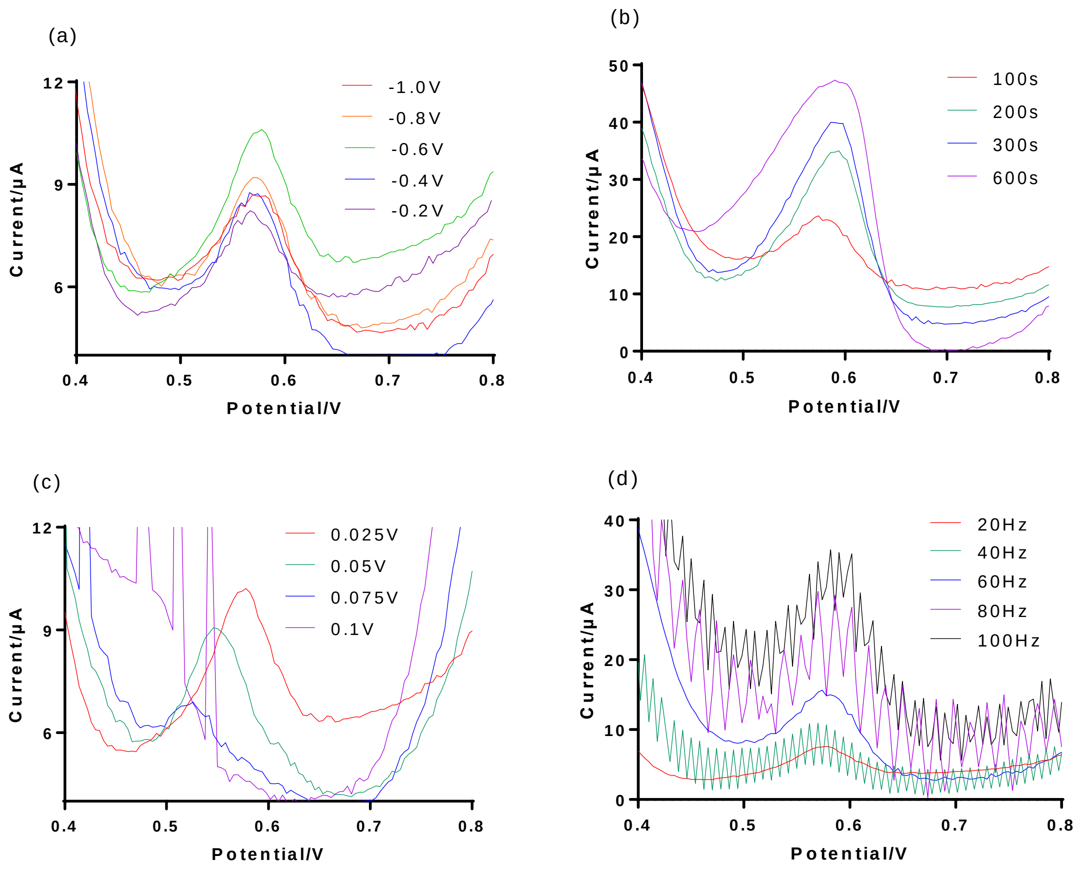


**Figure S3.** Effects of different **(a)** deposition potentials, **(b)** deposition times, **(c)** amplitudes, and **(d)** frequencies on the SWASV for $Hg^{2+}$ detection using an AuNP-biopolymer coated carbon SPE sensor.

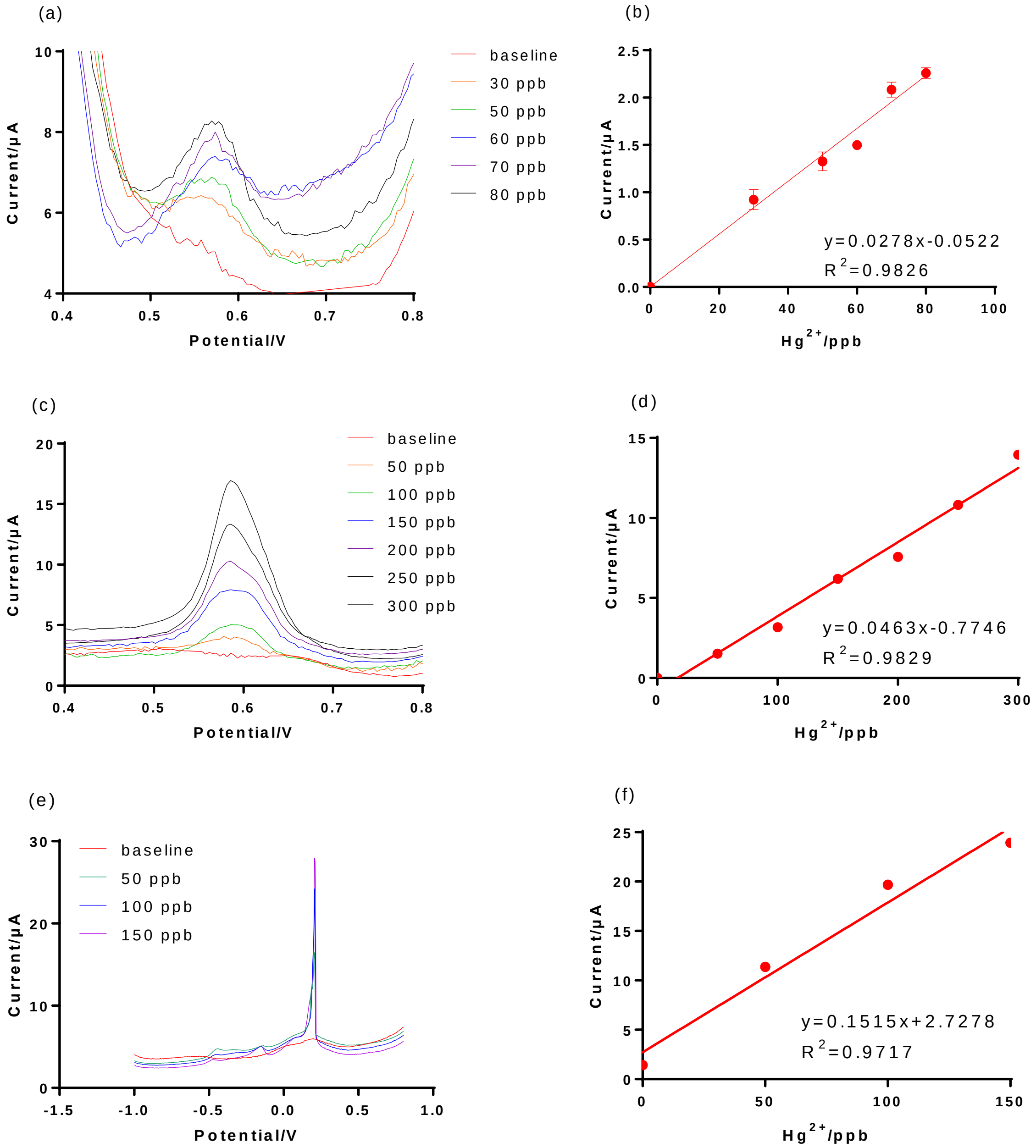


**Figure S4.** SWAVS for $Hg^{2+}$ detection and calibration curves before the parameter optimization. (**a** and **b**) AuNP-biopolymer coated carbon SPE sensor (prior to operational parameters optimization), (**c** and **d**) Bare gold SPE sensor, and (**e** and **f**) Bare carbon SPE sensor. Deposition time is 100 s with a -0.8V deposition potential, 0.004 V potential step, 25 mV amplitude, and 20 Hz frequency.

**Table S1.** Characterization of the landfill leachate

| Items | Concentration (mg/L) | Items | Concentration (mg/L) |
|---|---|---|---|
| pH | 7.73 | Thallium | 0.0007 |
| Total Phenolics | 4.63 | Lead (Pb) | 0.0028 |
| Zinc (Zn) | 0.052 | Mercury (Hg) | 0.00055 (0.55 ppb) |
| Boron (B) | 9.7 | Selenium (Se) | 0.022 |
| Chromium (Cr) | 0.31 | Cadmium (Cd) | 0.0016 |
| Cobalt (Co) | 0.08 | Total suspended solids (TSS) | 14 |
| Copper (Cu) | 0.028 | Total dissolved solids (TDS) | 8,870 |
| Lithium (Li) | 0.031 | Oil & Grease (petroleum/mineral) | 2.6 |
| Manganese (Mn) | 0.18 | Chloride (Cl) | 2,720 |
| Molybdenum (Mo) | 0.012 | Cyanide (CN) | 0.0313 |
| Nickel (Ni) | 0.27 | Total Kjeldahl Nitrogen (N) | 1,750 |
| Sodium (Na) | 2,430 | Total Phosphorus (TP) | 12.2 |
| Antimony (Sb) | 0.029 | Biochemical oxygen demand (BOD) | 764 |
| Arsenic (As) | 0.047 | Total Phenolics | 0.940 |
| Beryllium (Be) | 0.0007 | Conductivity (µS/cm) | 13,800 |
| Silver (Ag) | 0.0007 | | |
